# Resistant Hypertension and Obstructive Sleep Apnea

**DOI:** 10.1155/2013/193010

**Published:** 2013-05-28

**Authors:** Akram Khan, Nimesh K. Patel, Daniel J. O'Hearn, Supriya Khan

**Affiliations:** ^1^Division of Pulmonary and Critical Care Medicine, Oregon Health & Science University, Portland OR & Portland VA Medical Center, 3181 SW Sam Jackson Park Road, UHN67, Portland, Oregon 97239-3098, USA; ^2^Division of Nephrology and Hypertension, Oregon Health & Science University, Portland OR & Portland VA Medical Center, Portland, OR 97239, USA

## Abstract

Hypertension (HTN) is a modifiable, highly prevalent risk factor for cardiovascular morbidity and renal dysfunction worldwide. In the United States, HTN affects one in three adults, contributes to one out of every seven deaths and to nearly half of all cardiovascular disease-related deaths. HTN is considered resistant when the blood pressure remains above goal despite lifestyle modification and administration of three antihypertensive agents of different classes including a diuretic. Large population-based studies have suggested that obstructive sleep apnea (OSA) is a risk factor for resistant HTN. The mechanism proposed is a pattern of intermittent hypoxia associated with hyperaldosteronism, increased sympathetic tone, endothelial dysfunction, and inflammation. In this review we discuss the association between OSA and resistant HTN, the physiologic mechanisms linking OSA with resistant HTN, and the effect of continuous positive airway pressure therapy (CPAP) on blood pressure in patients with resistant HTN. While the reduction in blood pressure with CPAP is usually modest in patients with OSA, a decrease of only a few mmHg in blood pressure can significantly reduce cardiovascular risk. Patients presenting to a center specializing in management of hypertension should be screened and treated for OSA as a potentially modifiable risk factor.

## 1. Introduction

Hypertension (HTN) is a modifiable, highly prevalent, risk factor for cardiovascular morbidity and renal dysfunction worldwide and is increasing in incidence [1–3]. During 2005–2008, approximately 68 million (31%) United States (US) adults aged ≥18 years had HTN [2]. Of these adults, 48 million (70%) were receiving pharmacologic treatment and 31 million (46%) had their condition controlled. Poor control of HTN, which was noted in 38 million (54%) of adults with HTN, can be due to a multitude of reasons; these include dietary indiscretions with sodium or alcohol, nonadherence to a drug-treatment plan, inadequate dosages of medications, drug-induced hypertension, and secondary causes of hypertension [3, 4] as follows.

*Secondary causes of resistant hypertension:*

- obstructive sleep apnea
- renal parenchymal disease
- renal artery stenosis
- primary aldosteronism
- Cushing's disease
- hyperparathyroidism
- coarctation of aorta
- pheochromocytoma
- intracranial tumors.

HTN is considered resistant when the blood pressure (BP) remains above goal (≥140/90 mmHg) despite lifestyle modification and administration of three antihypertensive agents of different classes including a diuretic [4]. While the true prevalence of resistant hypertension is not known due to nonadherence or inadequately treated patients, in a population-based sample of approximately 16,000 adults in the US, the prevalence of resistant HTN was 8.9% among individuals with HTN and 12.8% in individuals treated with antihypertensive medications [5].

Obstructive sleep apnea (OSA) is the intermittent cessation (apnea) or decrease (hypopnea) of airflow during sleep leading to oxygen desaturation and/or arousal and is classified as mild, moderate, or severe based on the number of these flow-limited breathing events per hour, the apnea-hypopnea index (AHI): mild (AHI 5–<15), moderate (AHI 15–<30), and severe (AHI ≥30) [10]. OSA affects 2–4% of adults, is associated with increased cardiovascular risks and mortality, and increases with age and obesity [10–19]. The signs, symptoms, and consequences of OSA may be due to physiological changes from repetitive collapse of the upper airway leading to sleep fragmentation, hypoxemia, hypercapnia, changes in intrathoracic pressure, and increased sympathetic activity [10, 15, 20, 21]. OSA is an independent risk factor for HTN and the severity of HTN has been shown to increase with the severity of OSA [20–24].

In the Sleep Heart Health Study, a prospective population-based cohort study designed to study the effect of OSA, patients with apnea hypopnea index (AHI) >15 had three times the odds of having HTN at baseline compared to controls [25]. After adjusting for demographics and anthropometric variables, the odds ratio (OR) for HTN for those with AHI ≥30 was 1.37 (95% confidence interval (CI), 1.03–1.83;  *P* = 0.005) compared to those with AHI <1.5 [20]. In the Zaragoza Sleep Cohort Study, an observational study of patients referred for evaluation of sleep-disordered breathing with a median 12.2 years of followup, the adjusted hazard ratios (HR) for incident HTN were greater among patients who declined CPAP (1.96; 95% CI, 1.44–2.66), and those nonadherent to CPAP (1.78; 95% CI, 1.23–2.58), compared with OSA patients who were treated with CPAP (0.71; 95% CI, 0.53–0.94) [24]. Both these large population-based studies suggest an association between OSA and HTN. The mechanism proposed for the association between OSA and HTN is a pattern of intermittent hypoxia which leads to increased sympathetic tone, endothelial dysfunction, and inflammation leading to persistent elevation of blood pressure [20].

## 2. Resistant Hypertension and Obstructive Sleep Apnea

While population studies on the association between resistant HTN and OSA are lacking, multiple clinic-based studies have shown a significant association between resistant HTN and OSA [8, 26–29]. Resistant HTN and OSA share several modifiable risk factors including older age and obesity [4, 5, 30]. In a study by Logan et al., the prevalence of OSA defined as an AHI ≥10 was 65% in women and 95% in men in a cohort of 41 subjects with resistant HTN [26]. The same group of researchers subsequently showed that the treatment of subjects with CPAP lead to improvement in BP control [6]. Both studies used 24 hr ambulatory blood pressure (24 hr ABP) monitoring to assess HTN. In a case series by Gonçalves et al., OSA was present in 71% of cases and 38% of controls [31]. In this series, using a logistic regression model, OSA was found to be independently associated with resistant HTN (OR 4.8; 95% CI, 2.0–11.7).

In a case series by Gus et al., the risk for OSA as assessed by the Berlin Questionnaire was significantly increased in the subjects with resistant HTN as compared to those with HTN adequately controlled by medication [32]. In a similar study by Calhoun et al., 63% of subjects presenting to a resistant HTN clinic were identified to be at high risk for OSA using the Berlin Questionnaire [27]. The association of OSA with resistant HTN was confirmed by Pratt-Ubunama et al., who showed that OSA diagnosed by polysomnography was present in 85% of subjects with resistant HTN presenting to their [28]. Similarly, in an observational study of 125 consecutive subjects (age: 52 ± 10 years, 43% males, systolic and diastolic BP: 176 ± 31 and 107 ± 19 mmHg, resp.), OSA (AHI >15 events per hour) was the most common condition associated with resistant HTN (64.0%), followed by primary aldosteronism (5.6%), renal artery stenosis (2.4%), renal parenchymal disease (1.6%), oral contraceptives (1.6%), and thyroid disorders (0.8%) [29].

While these studies are based on case series and clinic-based cohorts, which are likely to have a higher prevalence of both resistant HTN and OSA, they suggest a strong association between the two conditions. 

## 3. Pathogenic Mechanisms Linking Resistant Hypertension with Obstructive Sleep Apnea

The major pathogenic mechanism linking resistant HTN with OSA may be hyperaldosteronism, although increased sympathetic tone, intermittent hypoxia, endothelin- and hypoxia-mediated vasoconstriction, and obesity may also play a role in the pathogenesis. We will next discuss these mechanisms in detail.

### 3.1. Hyperalderstronism

Aldosterone excess may play a pathophysiological role in the relation between HTN and OSA [27, 28, 33, 34]. Aldosterone assists in the regulation of circulating blood volumes and potassium concentration via a feedback loop within the adrenal cortex. Studies have shown that obese individuals have higher levels of aldosterone relative to their nonobese counterparts [35]. It has been proposed that increased obesity leads to an increase in aldosterone levels predisposing obese individuals to resistant HTN [35].

In an observational study of consecutive subjects with resistant HTN, subjects were evaluated for primary hyperaldosteronism using plasma rennin activity <1.0 ng/mL/hr and 24 hr urinary aldosterone excretion >12 micro g during high urinary sodium excretion (>200 mEq/24 hr) and risk of sleep apnea using the Berlin Questionnaire. Patients at high risk for OSA were almost two times more likely to have primary hyperaldosteronism (36% versus 19%, *P* < 0.05) with low plasma renin activity (1.2 ± 1.8 ng/mL/hr versus 1.9 ± 4.1 ng/mL/hr) and significantly greater 24 hr urinary aldosterone excretion (13.6 ± 9.6 mcg versus 9.8 ± 7.6 mcg, *P* < 0.05) compared to subjects at low risk of OSA, suggesting an association between primary hyperaldosteronism and OSA [27]. In another study by the same group, increased plasma aldosterone concentration and OSA were noted in patients with resistant HTN but not in control subjects, suggesting that aldosterone excess may contribute to OSA severity [28]. This group of researchers subsequently showed that OSA prevalence was significantly higher in subjects with hyperaldosteronism compared with hypertensive patients with normal aldosterone levels (84% versus 77%) [33]. Data from these studies suggests that hyperaldosteronism may be a key factor in linking resistant HTN with OSA. These studies are, however, all observational and causality cannot be directly established.

### 3.2. Increased Sympathetic Tone

Patients with OSA have high sympathetic activity when awake, with further increases in BP and sympathetic activity during sleep [21, 36]. These increases are attenuated by treatment with CPAP [36, 37]. The reasons for this high level of tonic sympathetic excitation are unclear, but may be linked to increased chemoreflex drive [38, 39]. Patients with OSA have been shown to have faster heart rates, decreased heart rate variability, and increased BP variability compared to controls [38, 39]. These abnormalities in cardiovascular variability have been linked to increased cardiovascular risk in OSA [21].

Catheter-based renal sympathetic denervation has been safely used to reduce renal efferent and possibly also afferent sympathetic activity and can substantially reduce BP in treatment-resistant hypertensive patients with normal renal function [40]. In a pig model of OSA, renal sympathetic denervation suppressed postapneic increase in BP and atrial fibrillation, suggesting a link between increased sympathetic tone, OSA, and resistant HTN [41]. These studies suggest the importance of the sympathetic tone in the pathogenesis of OSA however human studies would be needed to study the effect of renal sympathetic denervation on patients with OSA.

### 3.3. Endothelial Dysfunction and Intermittent Hypoxia

OSA is associated with intermittent decreases in oxygen saturation throughout the night. Endothelin, a potent vasoconstrictor, is released in response to hypoxia [42]. In animal models of intermittent hypoxia, there was a tendency for the animals exposed to intermittent hypoxemia to have systemic HTN [43, 44]. The proposed mechanism of the association between resistant HTN and OSA is that intermittent hypoxia from OSA leads to increase in endothelin production causing vasoconstriction; with subsequent reperfusion, endothelin production returns to normal, and the stimulus for vasoconstriction is abated. The cyclic changes in endothelin levels from intermittent hypoxia throughout the night in patients with OSA lead to development of resistant HTN [45, 46]. Endothelium-dependent vasodilation, measured by forearm blood flow after intra-arterial infusion of acetylcholine, an endothelium-dependent vasodilator, is reduced in healthy patients with severe OSA compared with age and body mass index matched controls [47, 48]. In a randomized trial of patients with OSA on CPAP therapy, the withdrawal of CPAP lead to impairment of endothelial function assessed by flow-mediated dilatation and increases in both systolic and diastolic BP and heart rate [49].

### 3.4. Obesity

Obesity, defined as a BMI ≥30, is a major risk factor for both uncontrolled HTN and OSA [4, 5, 10, 50]. In an analysis of Framingham Heart Study participants examined between 1990 and 1995, factors associated with poor BP control included older age, left ventricular hypertrophy, and obesity [51]. Obesity is also a common feature of patients with resistant HTN [4, 52]. Potential mechanisms of obesity-induced HTN include impaired sodium excretion, increased sympathetic nervous system activity, and the activation of the renin-angiotensin-aldosterone system [4].

### 3.5. Conclusion

In conclusion resistant HTN and OSA may have common pathogenic associations including hyperaldosteronism, increased sympathetic drive, endothelin- and hypoxia-mediated vasoconstriction, and obesity.

## 4. Role of Positive Airway Pressure Therapy in Treatment of Resistant Hypertension

OSA can be treated with positive airway pressure therapy, either CPAP or bilevel positive airway pressure (BPAP), oral appliance/mandibular advancement therapy, upper airway surgery, and tracheotomy [10]. CPAP is the most widely accepted form of therapy for OSA and remains the gold standard for treatment. A number of studies have examined the effect of CPAP on HTN. While there is significant amount of data on the effect of CPAP on HTN, data on resistant HTN is limited. Data from some of the key studies is summarized in Table 1. Two meta-analyses on the effect of CPAP on HTN showed modest effects [53, 54]. In the meta-analysis by Haentjens et al., 572 patients from 12 randomized controlled trials showed a net decrease of 1.69 mmHg in 24-hr mean ABP (95% CI, −2.69 to −0.69) [53]. While the meta-analysis by Alajmi et al. showed that the effects of CPAP were modest but were not statistically significant [54], CPAP (compared to control) reduced systolic BP (SBP) by 1.38 mmHg (95% CI: 3.6 to −0.88, *P* = 0.23) and diastolic BP (DBP) by 1.52 mmHg (CI: 3.1 to −0.07; *P* = 0.06). The impact was greater in individuals with severe OSA and improved with better CPAP compliance.

In a population-based study Marin et al. showed that in the Zaragoza Sleep Cohort Study treatment of OSA with CPAP was associated with a lower risk of associated HTN. Subjects were followed for a median of 12.2 years with a 37.3% incidence of HTN. Subjects with OSA ineligible for CPAP due to lack of sleepiness and AHI <15, or those declined or those who were noncompliant with CPAP had a higher incidence of HTN [24].

In a small study of 11 patients with resistant HTN by Logan et al., the use of nightly CPAP for two months was associated with a significant decrease in both systolic and diastolic BP [6]. Similarly, in a clinic-based study, Martinez-Garcia et al. showed that in 33 patients with difficult to treat HTN, compliance with CPAP reduced systolic BP, particularly at night, and normalized nocturnal BP patterns [7].

In a randomized prospective parallel trial, Lozano et al. randomized 75 patients with an AHI ≥15 to receive either CPAP and conventional treatment or conventional medical treatment alone. 24 hr ABP was repeated at three months. Patients who used CPAP ≥ 5.8 hr per night had clinically significant improvements in BP (−6.98 mmHg, 24 hr diastolic BP; −9.71 mmHg, 24 hr systolic BP) and also had a clinically significant improvement in nocturnal dipping pattern compared to the control group [9].

In a study of subjects with resistant HTN, Dernaika et al. used a retrospective cohort of subjects with OSA and resistant HTN (42 subjects) and controlled HTN (56 subjects) and showed that the use of CPAP permitted deescalation of antihypertensive treatment in 71% of subjects with resistant HTN but did not significantly alter the antihypertensive regimen in the control group [8]. Multivariate regression analysis showed that baseline BP (OR 5.4, 95% CI 2.3 to 8.9; *P* = 0.01) and diuretic therapy (OR 3.2, 95% CI 1.8 to 6.1; *P* = 0.02), but not AHI or hours of CPAP use, were independently associated with a decrease in mean arterial pressure after 12 months of CPAP therapy [8].

The evidence from these studies suggests that treatment of OSA with CPAP leads to modest reduction in BP in individuals with resistant HTN. This is significant because a decrease of only a few mmHg in BP is enough to significantly reduce cardiovascular risk [3].

## 5. Conclusions and Recommendations for the Practicing Physician

HTN is highly prevalent in the adult population; up to 31% of adults >18 years old in the United States have been found to have high BP. Resistant HTN, when the BP remains above goal (≥140/90 mmHg) despite lifestyle modification and administration of three antihypertensive agents of different classes, can be present in up to 8.9% of individuals with HTN and 12.8% of individuals on antihypertensive medications. OSA is present in 2% to 4% of adults and is a common modifiable risk factor for resistant HTN. OSA may predispose predispose to resistant HTN via hyperaldosteronism, obesity, increased sympathetic drive, endothelin, or hypoxic vasoconstriction.

Studies have shown that treatment of OSA with CPAP can lead to improvement in BP control in patients with resistant HTN. While the reduction is usually modest, a decrease of only a few mmHg in BP can significantly reduce cardiovascular risk. Patients presenting for the management of resistant HTN should be screened for OSA as a modifiable risk factor. Referral to a sleep center can help improve BP control by early diagnosis and treatment of OSA when present, and improving compliance with CPAP in individuals who have OSA.

## Figures and Tables

**Table 1:** Effects of continuous positive airway pressure therapy on resistant hypertension.

Study	Study design, population	Sample size (*n*)	Intervention	Results
Logan et al., 2003 [6]	Prospective observational, refractory HTN, AHI ≥ 10	11	CPAP for 2 nights in sleep lab and then 2 months	CPAP used ↓ BP acutely and over two months

Martinez -Garcia et al., 2007 [7]	Prospective observational, difficult to treat HTN, AHI ≥ 15	33	CPAP for 2 months	CPAP ↓ systolic BP, particularly at night

Dernaika et al., 2009 [8]	Retrospective chart review, observational, HTN, AHI > 5	98	CPAP therapy for 1 year, controlled HTN (*n* 56) versus resistant HTN (*n* 42)	CPAP permitted deescalation of anti-HTN treatment in 71% of subjects with resistant HTN, no significant change in controlled HTN

Lozano et al., 2010 [9]	Prospective randomized controlled parallel trial, resistant HTN, AHI ≥ 15	75	CPAP (*n* 38) versus conventional treatment (*n* 37)	CPAP for 3 months led to ↓ in 24 hr ABP in patients who used CPAP > 5.8 hr/night

24 hr ABP: 24-hour ambulatory blood pressure monitoring, AHI: apnea hypopnea index, BP: blood pressure, CPAP: continuous positive airway pressure, HTN: hypertension.
